# Frequency, Timing, Burden and Recurrence of Adverse Events Following Immunization After HPV Vaccine Based on a Cohort Event Monitoring Study in the Netherlands

**DOI:** 10.3390/vaccines13080812

**Published:** 2025-07-30

**Authors:** Monika Raethke, Jeroen Gorter, Rachel Kalf, Leontine van Balveren, Rana Jajou, Florence van Hunsel

**Affiliations:** 1 Netherlands Pharmacovigilance Centre Lareb, Goudsbloemvallei 7, 5237 MH ’s-Hertogenbosch, The Netherlands; 2 Department of PharmacoTherapy—Epidemiology & Economics, Groningen Research Institute of Pharmacy (GRIP), University of Groningen, Broerstraat 5, 9712 CP Groningen, The Netherlands

**Keywords:** HPV vaccination, Adverse Events Following Immunization (AEFI), Dutch National Immunization Program, cohort event monitoring (CEM)

## Abstract

**Background/Objectives:** The aim of this study was to systematically assess Adverse Events Following Immunization (AEFI) among children following administration of the human papillomavirus (HPV) vaccine (Cervarix^®^) included in the Dutch National Immunization Program (NIP) and to characterize the pattern and recurrence risk of AEFI after HPV revaccination. **Methods:** A longitudinal cohort event monitoring study, using patient-reported outcomes was used among recipients of the HPV vaccine at 10 years of age. Data were available for 3063 children following the first HPV vaccination and for 2209 children following the second HPV vaccination. **Results:** The most commonly reported AEFI following HPV vaccination were injection site reactions—reported by 46.5% of participants after the first dose and 31.9% after the second dose—followed by headache (8.2% and 3.9%, respectively) and joint pain (4.5% and 3.7%, respectively). Participants who received both HPV vaccine doses reported more AEFI after the first dose than after the second. Among girls, 61.2% reported at least one AEFI following the first dose, compared to 44.2% after the second dose. For boys, these percentages were 55.3% and 38.5%, respectively. This difference was statistically significant (*p* = 0.002). For some AEFI, such as injection site reactions, there appears to be a potential increased risk of recurrence following the second dose. **Conclusions:** This prospective longitudinal cohort event monitoring study showed that AEFI were more frequent after the first HPV dose and more frequent for girls compared to boys. An increased risk of recurrence was seen for AEFI, such as injection site reactions and headache. Furthermore, this study provides insight into the course of AEFI and the extent to which children were affected by these symptoms based on real-world data.

## 1. Introduction

The Dutch National Immunization Program (NIP) has provided protection for children in the Netherlands against serious infectious diseases for nearly 70 years. The immunization schedule commences in infancy, with the final routine vaccination scheduled at the age of 14. This schedule is periodically revised in response to the introduction of new vaccines or emerging scientific insights [1]. Adjustments to the schedule may warrant enhanced monitoring of the safety profile of the vaccines administered in the subsequent period.

In 2009, the human papillomavirus (HPV) vaccination was first offered to girls born between 1993 and 1996 as part of a catch-up campaign. Since 2010, HPV vaccination has been incorporated into the NIP for girls at the age of 12 [2]. Initially, three doses were required; however, in 2014 this was revised to a two-dose regimen for girls until the age of 14. From mid-2022 this dose regimen was used for all ages. Given that the vaccine is most effective when administered prior to exposure to HPV and benefits both girls and boys, as of January 2022, the HPV vaccine has been offered to all children (both girls and boys) during the year in which they turn 10 years old [3]. In addition, catch-up campaigns were conducted from 2022 to June 2024 targeting adolescents and young adults up to the age of 26 [4].

Within the NIP, the Cervarix^®^ vaccine is used. This bivalent vaccine offers protection against persistent HPV infection and (precursors of) HPV-related cancers caused by HPV types 16 and 18. These include cervical cancer and cancer in other parts of the body—the mouth, throat, vagina, labia, penis and anus. Full protection requires two doses [5]. There is also evidence that vaccination has cross-protective effects against other HPV types such as HPV 31, 33, and 45 [6,7]. Vaccination also helps to protect the entire population by creating herd immunity, which makes it harder for the virus to spread [6,7].

The National Institute for Public Health and the Environment (RIVM) in the Netherlands previously conducted research on potential adverse events following HPV vaccination. To evaluate the tolerability of the catch-up campaign, adverse events occurring within seven days post-vaccination with the bivalent HPV vaccine were assessed. For this study, 6000 girls were invited—1500 per birth cohort (1993–1996). After each of the three vaccine doses, participants received an online questionnaire addressing local and systemic reactions. A total of 4248 girls completed at least one questionnaire. The results indicated that local reactions such as arm pain were frequently reported: 92.1% after the first dose, 79.4% after the second, and 83.3% after the third. Systemic adverse events, including myalgia, were reported by 91.7%, 78.7%, and 78.4% of participants following the first, second, and third doses, respectively. The incidence of adverse events was lower after subsequent doses. Additionally, older participants reported adverse events more frequently than younger ones [2].

Lareb serves as the Dutch national pharmacovigilance center, responsible for monitoring adverse events related to medicinal products, including vaccines, as well as drug use during pregnancy and breastfeeding [8]. With this HPV Cohort Event Monitoring study, Lareb aims to systematically assess Adverse Events Following Immunization (AEFI) among children following administration of the HPV vaccine included in the NIP. The expansion of the vaccination program to include boys and lowering the age underscored the necessity for additional surveillance and monitoring.

The aim of this study is to characterize the characteristics and recurrence risk of AEFI after HPV vaccination. The following research questions were drafted as sub-aims:

I. What types of AEFI occur, at what frequency, and following which vaccination moment?
II. Are there serious AEFI reported post-HPV vaccination that necessitate medical treatment?
III. What is the time of onset and duration of reported AEFI?
IV. Are there identifiable risk factors associated with AEFI?
V. What is the likelihood of experiencing similar AEFI following a subsequent HPV vaccination, i.e., the recurrence risk?

## 2. Materials and Methods

### 2.1. Study Design and Population

This study was conducted as part of a Lareb vaccine monitoring project using the Lareb Intensive Monitoring (LIM) tool, adapted for use in this particular population [9] and employed a longitudinal cohort design with Patient Reported Outcomes (PROs). Children aged ±10 years were followed from the time of their first HPV vaccination through to their second HPV vaccination. Following each vaccination, parents or legal guardians completed questionnaires assessing the presence or absence of AEFI.

Eligible participants were parents/guardians over the age of 16 whose child was born in 2013, 2014, or 2015, and who intended to follow the Dutch NIP. Participants had to be proficient in Dutch and have access to a computer, tablet, or smartphone with internet access to complete the digital questionnaires. Only residents of the Netherlands were eligible.

**Figure 1:**
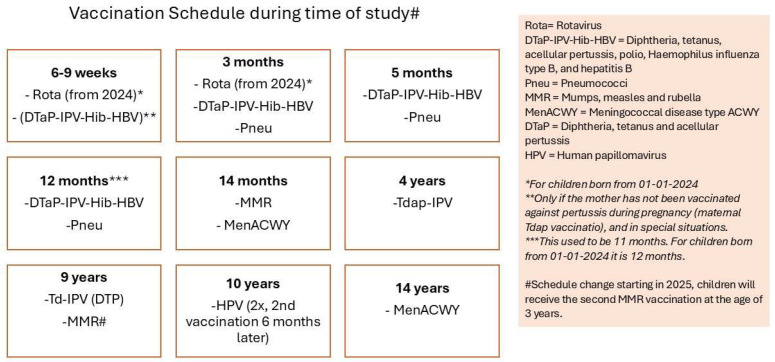
Vaccination schedule of the NIP in the Netherlands, including HPV [10].

The study period spanned from August 2022 to December 2024. The complete vaccination schedule of the Dutch NIP is shown in Figure 1, which also includes the HPV vaccination schedule of this study.

### 2.2. Ethics Approval

If a study in the Netherlands is subject to the Medical Research Involving Human Subjects Act (WMO), it must undergo a review by an accredited Medical Research Ethics Committee or the central committee on research involving human subjects (CCMO). After submission to an accredited review committee (METC Brabant, review number NW2022-12), this CEM study was deemed not to fall under the WMO. No ethics approval was needed for this study.

### 2.3. Recruitment and Study Entry

Eligible parents/guardians received an invitation letter, sent via the infrastructure of the RIVM, specifically through its Vaccine Supply and Prevention Programmes (DVPs). The letter included a flyer listing the project website (https://www.hpvmonitor.nl/), where detailed study information, including a privacy statement, study protocol, and FAQs were available. During registration, informed consent was obtained digitally. Upon confirmation of participation, parents/guardians received digital questionnaires regarding AEFI following each of the two monitored vaccination moments.

### 2.4. Questionnaire Timing

For the HPV vaccine, AEFI were expected within 48 h, with resolution typically within 1–2 days [5]. The first post-vaccination questionnaire was sent one week after HPV dose 1, followed by a second follow-up questionnaire two weeks later. This scheduling was necessary to provide participants with sufficient time to register for the study and complete the questionnaires. However, the delay between the occurrence of most AEFI and the timing of the questionnaire could potentially lead to recall bias. Participants who had not yet completed the first questionnaire received an automated reminder 2 and 5 days after the questionnaire was sent. The first questionnaire for the second dose was scheduled to be sent 210 days after the first dose with subsequent reminders for those participants who had not responded yet. 

### 2.5. Study Withdrawal

Participation ended under one of the following conditions: (1) completion of the final questionnaire, (2) active withdrawal by the participant at any time without the need for justification, or (3) failure to complete the final questionnaire within the allowed timeframe.

### 2.6. Data Collection

The questionnaires captured demographic and health-related characteristics of the child, including sex, age, comorbidities, medication use, number of children in the household, parental education level (7-levels based on Dutch qualification levels), use of after-school care, receipt of vaccinations outside the NIP, and any AEFI and its impact.

Participants were presented with a checklist of common, known adverse events—such as injection site reactions, headache, fever, nausea, vomiting, joint pain, and rash—as well as an open-text field for less common or unexpected AEFI. They were also asked whether the AEFI led to hospital admission and whether antipyretic medication was used either prophylactically or therapeutically.

Information was collected on the time to onset (TTO) and duration (in hours) of AEFI, and severity of the complaint, rated on a five-point scale ranging from “not bothersome” to “very burdensome.”

### 2.7. Data Analysis

All AEFI from the questionnaires were coded using the preferred terms (PTs) from the Medical Dictionary for Regulatory Activities (MedDRA, version 26.1) [11]. Seriousness of AEFI was coded according to the Council for International Organizations of Medical Sciences (CIOMS) criteria [12]. A formal causality assessment was not performed, all reported AEFI were included.

Summary data were presented in tables showing the proportion of participants reporting AEFI for each vaccination event. In addition, we assessed sex differences in the occurrence of AEFI. Statistical significance was determined using Chi-square tests (*p* < 0.05 was considered statistically significant), and results were visualized in a heatmap stratified by dose. TTO and duration of AEFI were visualized with boxplots. The burden was visualized in a stacked bar chart. 

### 2.8. Calculation of Recurrence Risk

As HPV vaccination involves two doses, the risk of experiencing the same AEFI after both doses was calculated. This recurrence risk was defined as follows:

Recurrence:

             *Risk of a specific AEFI after both dose 1 and dose 2*

*Risk of a specific AEFI not occurring after dose 1, but AEFI occurring after dose 2*

**Figure 2:**
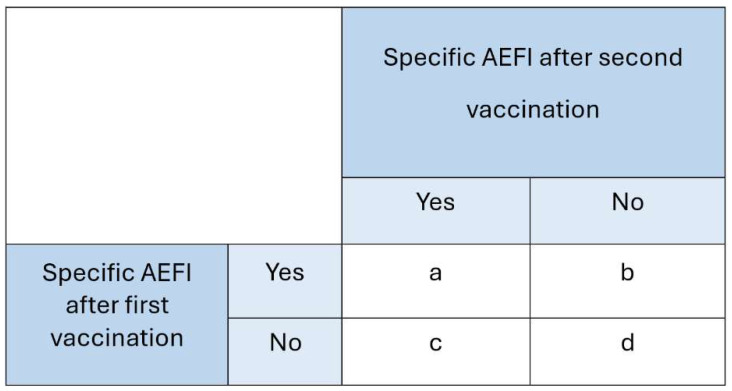
Calculation for the risk ratio.

The risk ratio (RR) was computed to quantify the strength of association between vaccination dose and the recurrence of AEFI using the variables defined in Figure 2.

The following calculation was used for the recurrence risk analysis:

**Numerator**: a/(a+b)— the risk among participants who reported a specific AEFI after the first HPV dose and experienced the same AEFI again after the second dose.
**Denominator**: c/(c+d)— the risk among participants who did not report a specific AEFI after the first HPV dose but did experience it after the second dose.

Several potential influencing factors were included in the recurrence risk analysis: the child’s sex, attendance at after-school care, and whether the child lived in a household with multiple children. These factors were incorporated into a multivariable logistic regression model to estimate the association with recurrence risk, with results presented as odds ratios and 95% confidence intervals.

To ensure reliable estimation of recurrence risk, calculations were only performed for specific AEFI that were reported by at least 10 children after the second vaccination. If a participant reported experiencing the same AEFI multiple times following a single vaccination, the AEFI was only counted once in the analysis.

All analyses were performed using R statistical software (version 4.4.2). 

## 3. Results

### 3.1. Number of Participants and Baseline Characteristics

**Figure 3:**
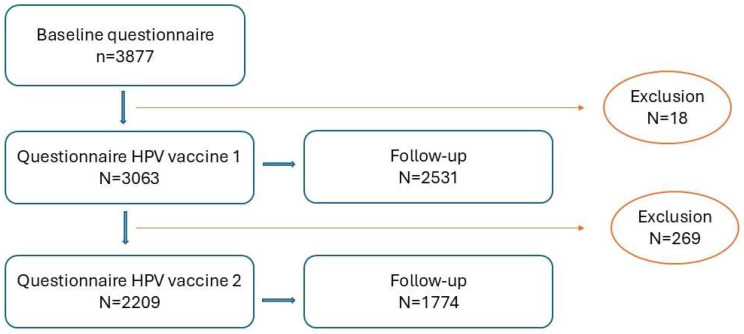
Participant flowchart of the study.

The baseline questionnaire was filled in by 3877 participants. Subsequent questionnaires for HPV vaccination 1 and 2 were filled in by 3063 (79%) and 2209 (57%) of participants. For each questionnaire, there was a follow-up, to capture data on AEFI with a longer TTO or duration. The participant flow-chart of the study is presented in Figure 3. In the flowchart, attrition is based on participant drop-out and from exclusion if participants did not fit the inclusion criteria for this study. 

The characteristics of the children for whom parents or guardians completed the intake questionnaire, as well as the questionnaires for HPV dose 1 or HPV dose 2, are presented in Table 1. The distribution of boys and girls in the cohort is approximately equal. Approximately 6.8% of the children for whom questionnaires were completed were reported to have an underlying medical condition. The reported conditions are categorized and presented by organ system, in which respiratory, thoracic and mediastinal disorders were most frequently reported (1.8%).

Table 2 presents the household characteristics of the families of participating children. The majority of children come from two-child households (59.6% for HPV 1 and 60.3% for HPV2). Larger families (>3 children) were observed but represented a minority within the study population. Parents of participating children were generally highly educated: 43.8% had completed a degree in higher professional education and 37.7% held at least a university degree. Approximately half of the children attended after-school care.

### 3.2. Reported Adverse Events Following Immunization (AEFI)

Table 3 presents the number of participants who reported one or more AEFI for their child. Among participants who completed at least one questionnaire following the baseline and first HPV vaccination, 1812 (59.2%) reported experiencing at least one AEFI. Following the second HPV vaccination, this figure was 914 (41.4%). None of the reported AEFI were defined/considered serious according to CIOMS seriousness criteria.

**Figure 4:**
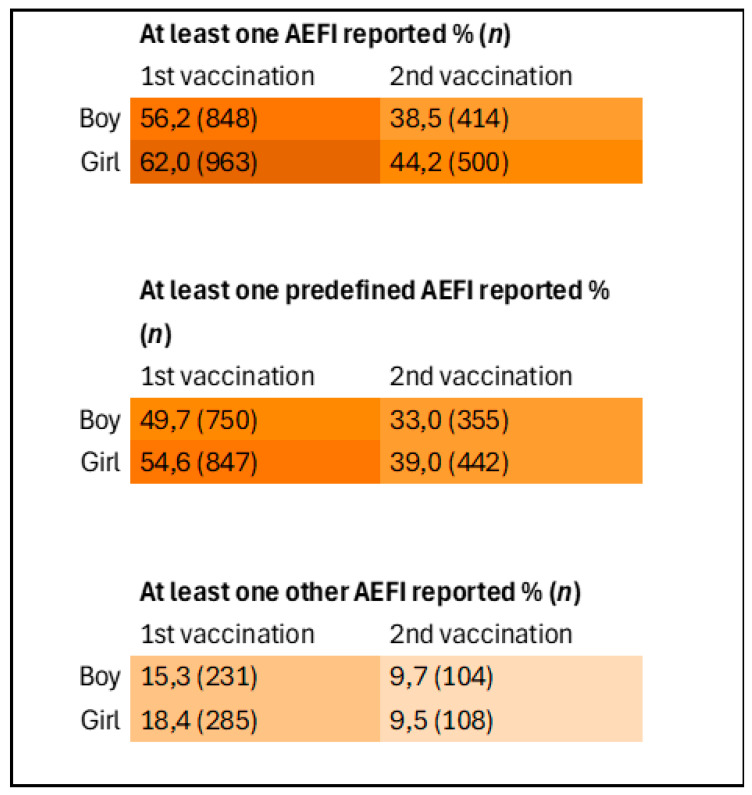
Heatmap of reporting of AEFI stratified by sex and vaccination moment. Inclusions 1st vaccination: boys *n* = 1508, girls *n* = 1552. Inclusions 2nd vaccination: boys *n* = 1075, girls = 1132.

Figure 4 presents a heatmap showing the differences in AEFI reporting between boys and girls following the first and second HPV vaccination. A statistically significant difference was observed in the proportion of participants reporting at least one AEFI, with girls’ parents reporting AEFI more frequently than boys’ parents. This difference was significant both across both vaccination moments combined (Chi-square test, *p* = 0.002), as well as when analyzed separately for the first vaccination (Chi-square test, *p* = 0.002) and the second vaccination (Chi-square test, *p* = 0.002).

The reported AEFI primarily consisted of local reactions at the injection site, such as pain or swelling. These were reported by 1424 (46.5%) of participants following the first vaccination and 705 (31.7%) following the second vaccination. In addition, AEFI such as headache (*n* = 252, 8.2%) and arthralgia (*n* = 137, 4.5%) were also occasionally reported. An overview of these complaints is presented in Table 4.

In addition to the pre-defined AEFI listed in the questionnaire, other AEFI were also reported, including fatigue (3.0% after the first vaccination and 1.1% after the second vaccination), myalgia (2.9% after the first vaccination and 2.7% after the second vaccination), and limb pain (2.7% after the first vaccination and 1.7% after the second vaccination). An overview of the top 10 reported complaints is presented in Table 5.

No hospitalizations were required for any of the reported AEFI. Beyond the top 10 shown in Table 5, there was one case of herpes zoster following the second vaccination. It was reported that symptoms began three weeks after vaccination, with recovery occurring 10 days after the symptoms started. The child was also treated with medication. Additionally, two children experienced cold sores (oral herpes/herpes labialis): parents of one child reported it two weeks after the first HPV vaccination, and the parents of the other child reported it one week after the second vaccination. A few children also reported nosebleeds, which began five minutes, three days, and five days after the HPV vaccinations, respectively. One child experienced frequent nosebleeds since the vaccination. Two children experienced unexpected reactions: one child had an increased allergic reaction to a mosquito bite compared to usual, and another child reported pain at the injection site of the previous HPV vaccination one week after the second vaccination. An overview of all reported complaints is included in the Supplementary Materials.

### 3.3. Time to Onset, Time to Recovery and Burden of AEFI

**Figure 5:**
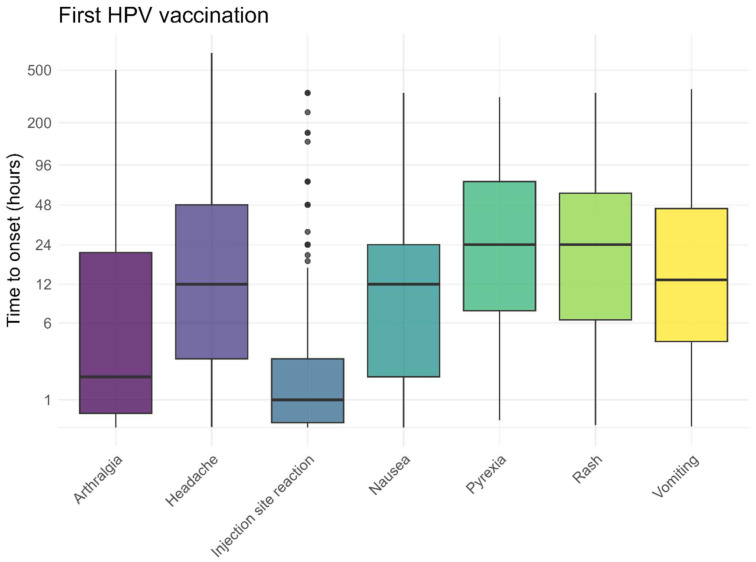
Time to onset for solicited AEFI after the first HPV vaccination, dots represent outliers.

**Figure 6:**
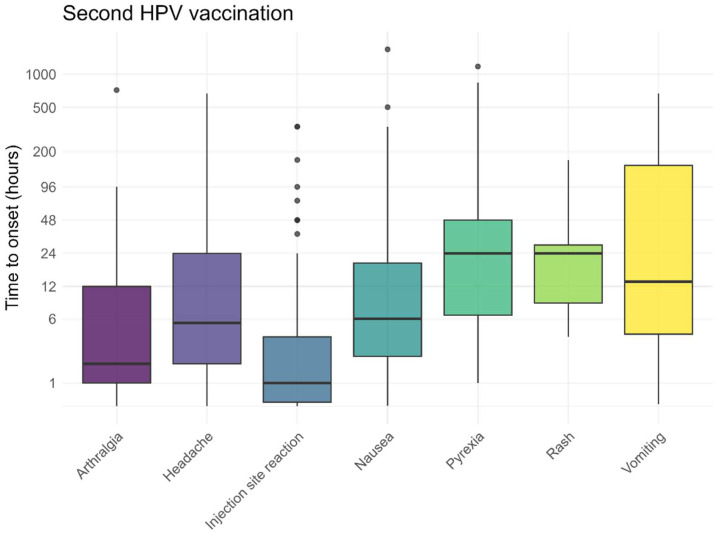
Time to onset for solicited AEFI after the second HPV vaccination, dots represent outliers.

The pre-defined AEFI generally occurred within 48 h after vaccination, both following the first and second HPV vaccinations (Figure 5 and Figure 6). Local reactions at or around the injection site, such as redness, pain, or swelling, typically developed shortly after vaccination (median 1 h). As shown in Figure 5 and Figure 6, some of these reactions occurred within the first hour, including the pain experienced at the time of injection. The time to onset for other, non-solicited, AEFI is presented in the Supplementary Materials. 

**Figure 7:**
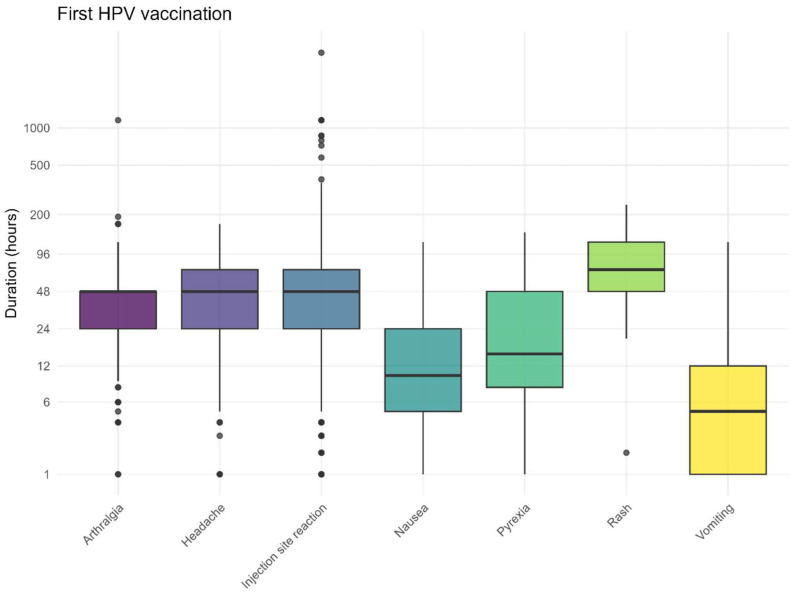
Duration for solicited AEFI after the first HPV vaccination, dots represent outliers.

**Figure 8:**
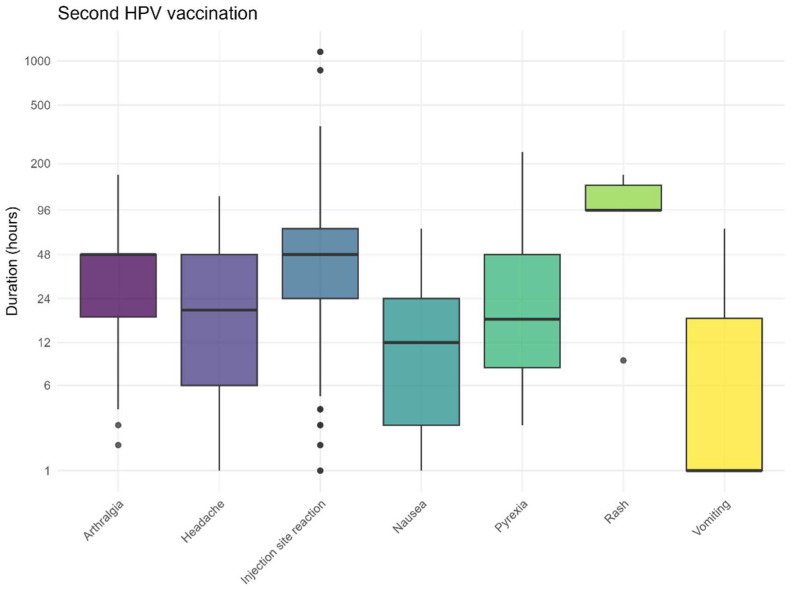
Duration for solicited AEFI after the second HPV vaccination, dots represent outliers.

The recovery times followed the expected pattern, with the most frequently reported (solicited) AEFI resolving after approximately 3 days (Figure 7 and Figure 8). The recovery from skin rashes took slightly longer than that for other reported AEFI, both after the first and second vaccination. For injection site reactions, some children experienced prolonged symptoms. The recovery times for non-solicited AEFI are also provided in the Supplementary Materials.

**Figure 9:**
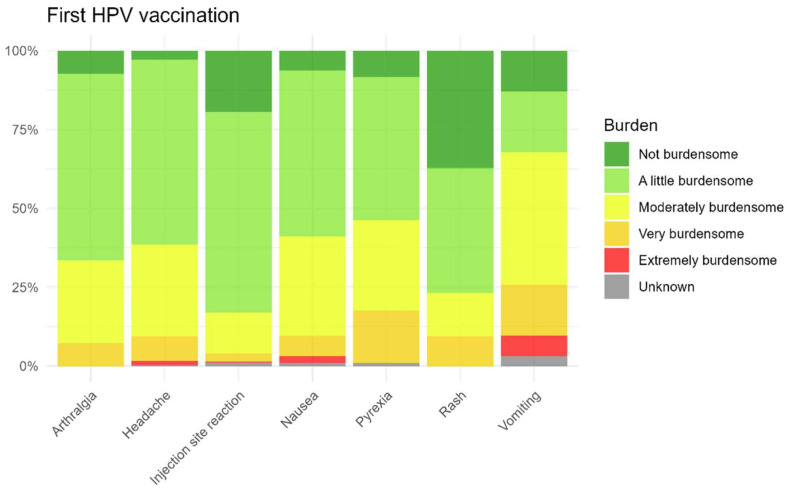
Burden for solicited AEFI after the first HPV vaccination.

**Figure 10:**
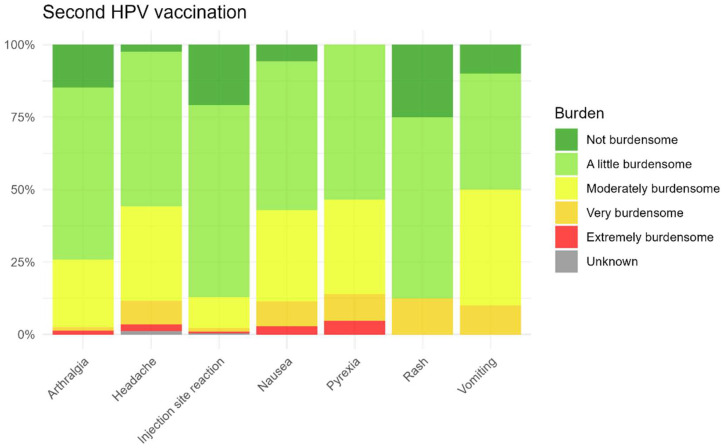
Burden for solicited AEFI after the second HPV vaccination.

As shown in Figure 9 and Figure 10, parents of the children generally reported the most commonly reported AEFI after the first and second HPV vaccinations as non-burdensome to slightly burdensome for their child. Among the most commonly reported AEFI, vomiting was perceived as the most burdensome, but only after the first injection, while injection site reactions were considered less burdensome after both vaccinations. The perceived burden for non-solicited AEFI is also presented in the Supplementary Materials.

### 3.4. Recurrence Risk

Injection site reactions were the most frequently reported AEFI in this study. Approximately 47% of participants reported this reaction after the first vaccination, and around 32% reported it after the second vaccination. In determining the recurrence risk, the relative risk of experiencing the same AEFI at both vaccination moments was assessed. Injection site reactions were reported by 499 participants at both vaccination moments. The likelihood of reporting this reaction at both times was significantly increased (RR = 2.88 with 95% confidence interval: 2.51–3.31). For injection site reactions, the sex of the child was found to influence the recurrence risk. Boys had a lower relative risk of experiencing an injection site reaction at both vaccination moments compared to girls (RR = 0.84 with 95% confidence interval 0.75–0.95). Out-of-school care attendance or having multiple children in the household did not affect the recurrence risk for injection site reactions.

Headache, the second most reported complaint after injection site reactions, was reported by about 8% of participants after the first vaccination and by approximately 4% after the second vaccination. In determining the recurrence risk, the relative risk of reporting the same AEFI at both vaccination moments was assessed. Headache was reported by 24 participants at both vaccination moments. Despite the small absolute number, the likelihood of reporting a headache at both times was increased (RR = 5.34 with 95% confidence interval 3.42–8.33). The child’s sex, out-of-school care attendance, or multiple children in the household did not significantly contribute to the recurrence risk for the reported headaches.

The recurrence risks for other reported AEFI are provided in the Supplementary Materials.

## 4. Discussion

Vaccines offer protection against infectious diseases by stimulating an immune response within the body, leading to the production of immune cells and antibodies specific to a pathogen. Although vaccines can cause adverse reactions, the majority of these adverse reactions occur relatively soon after administration and are primarily mediated by the innate immune system’s response to the vaccine. These reactions include local inflammatory responses, such as redness, pain, and swelling at the injection site, as well as systemic symptoms such as fever and headache [13,14]. A more comprehensive understanding of the symptom profile associated with current NIP vaccinations could enhance the quality of information regarding potential vaccine-related adverse effects.

This study included a cohort of children who were followed after receiving the first and second HPV vaccinations. Data were available for 3063 children following the first HPV vaccination and for 2209 children following the second HPV vaccination. After the first HPV vaccination, 59.2% of participants reported experiencing at least one suspected AEFI, whereas 41.4% reported AEFI following the second HPV vaccination. This percentage is lower than that previously found in a cohort of Dutch girls receiving HPV vaccination [2]. A difference with our study is an older age at which the vaccination was given, 13 to 16 years vs. 10 years in our study. Also, questionnaires in the study by van Klooster et al. [2] were filled in by girls themselves, while they were completed by parents or caregivers in our study. The type of AEFI observed in our study aligns with findings from previous studies [2,14,15]. Additionally, the girls’ parents reported significantly more AEFI than boys.

In prior studies, age, vaccination moment, vaccine batch, and needle type have been identified as risk factors for the occurrence of common AEFI [16,17,18], while sex in children (unlike in adults) did not appear to be a significant factor [19]. In contrast, cohort studies in adults have demonstrated clear differences in AEFI occurrence based on sex, with a higher percentage of AEFI reported among women [20,21]. There are clear sex differences in immune responses between men and women [22,23,24]. It is hypothesized that hormonal changes at the time of the HPV vaccination may contribute to the observed differences in incidence of AEFI between boys and girls. Conducting analyses in younger age groups within similar cohort event monitoring studies in the Netherlands may offer deeper insights into this theory and help identify at which age sex differences begin to occur. However, it cannot be fully excluded that boys and girls talk about AEFI differently with their parents or caregivers and that this is subsequently reflected in differences in the reporting pattern. 

Prior experience of an AEFI is a known barrier to vaccination [25]. Given that the HPV vaccination involves two distinct administration moments, this study provided an opportunity to assess whether there is an increased risk of recurrence for previously reported AEFI. A number of AEFI, such as injection site reactions, headache, joint pain, muscle pain, nausea, and fever, were found to have an increased risk of recurrence. Informing parents on the characteristics of reported AEFI, such as TTO, duration and burden, as well as the potential risk of recurrence, supports informed decision making.

It is important to consider potential influencing factors, such as sex or genetic factors, in understanding these recurrence patterns. Although, not always significant, girls often had a higher risk for reoccurrence of AEFI than boys. A previous Australian study found that revaccination is safe for the majority of children with a personal or family history of AEFI [23]. A Canadian study estimated the risk of recurrence of AEFIs upon revaccination among patients (91% <18 years) with suspected vaccine allergy. Most individuals with previous AEFIs were safely revaccinated [26]. However, it is essential to note that for many AEFI in our current study, the sample sizes were small, which resulted in wide confidence intervals when calculating risk ratios.

### Strengths and Limitations of the Study

The Netherlands Pharmacovigilance Centre Lareb has extensive experience with cohort event monitoring through a custom-built web application, which facilitates the collection of PROs. This method is particularly well suited for monitoring common and recognizable AEFIs, which may not require contact with healthcare professionals. As such, patients or consumers are well placed to reliably report these experiences themselves, enabling the capture of valuable real-world data directly from vaccine recipients [27]. 

The use of PROs allows for a broad and timely overview of vaccine tolerability, particularly for reactogenic effects that might otherwise go unreported in traditional healthcare settings. It should be noted that the reported symptoms may not necessarily have a causal relationship with the administered vaccines; a potential overestimation of the reported events should be considered. Furthermore, assessing the recurrence risk may inadvertently imply there is a causal link between (re)vaccination and the reported events. This is not necessarily the case and should be carefully considered when interpreting the results. Clearly communicating this nuance is especially important when sharing findings with the general public. In cases where serious outcomes are reported, Lareb requests additional follow-up when necessary, including hospital discharge letters or medical documentation, to evaluate the association between the vaccination and the adverse event. However, in this study, no serious AEFI were reported.

Overall, this approach highlights the significant contribution of patient-reported outcomes to pharmacovigilance, offering timely, detailed, and patient-centered insights that complement data from other surveillance systems. 

The participant group in this study is not fully representative of all children enrolled in the Dutch Immunization Program. This is partly due to the inclusion criteria, which required participants to have a sufficient level of proficiency in the Dutch language. Additionally, the educational level of the parents or caregivers who completed the questionnaires was higher on average compared to the general population in the Netherlands [28]. Higher educational level has been associated with greater health awareness, which may contribute to a healthy vaccinee effect among the children who were enrolled in the study. Participation in the study and attrition during the follow-up period may have been selective. It is anticipated that participants with AEFI were more likely to complete the questionnaires. Furthermore, it is possible that some participants who missed questionnaires may no longer be enrolled in the NIP. It should be considered possible that participants discontinued due to their child experiencing a serious AEFI. Selective attrition may also influence the estimated symptom time course. To address this, further sensitivity analyses will be conducted to assess the impact of attrition on study outcomes.

## 5. Conclusions

In this prospective longitudinal cohort event monitoring study on AEFI after HPV vaccination, no events were reported that would indicate new or concerning adverse effects. More AEFI were reported after the first dose than after the second dose. Girls reported AEFI significantly more often than boys following HPV vaccination. For several specific AEFI, such as injection site reactions and headaches, an increased relative risk of experiencing this AEFI after both doses was observed. Furthermore, this study provides insight into the course of AEFI and the extent to which children were affected by these symptoms based on real-world data.

## Figures and Tables

**Table 1:** Characteristics of participants after baseline and questionnaire 1.

Characteristics Participants		
	HPV Dose 1 (*n* = 3063)	HPV Dose 2
		(*n* = 2209)
**Questionnaires filled in by unique participants, *n* (%)**		
Baseline questionnaire	3877	3877
First questionnaire (7 days after vaccination)	3063 (100)	2209 (100)
Follow-up questionnaire (14 days after vaccination)	2531 (82.6)	1774 (80.3)
**Sex, *n* (%)**		
Boy	1508 (49.2)	1075 (48.7)
Girl	1552 (50.7)	1132 (51.2)
Other *	3 (0.1)	2 (0.1)
**Birth cohort, *n* (%)**		
2013	313 (10.2)	136 (6.2)
2014	2733 (89.2)	2070 (93.7)
2015	17 (0.6)	3 (0.1)
**Existing medical conditions (SOC level), *n* (%)**		
Respiratory, thoracic and mediastinal disorders	55 (1.8)	39 (1.8)
Congenital, familial and genetic disorders	42 (1.4)	25 (1.1)
Immune system disorders	35 (1.1)	25 (1.1)
Skin and subcutaneous tissue disorders	27 (0.9)	18 (0.8)
Nervous system disorders	22 (0.7)	15 (0.7)
Gastrointestinal disorders	18 (0.6)	16 (0.7)
Psychiatric disorders	16 (0.5)	12 (0.5)
Metabolism and nutrition disorders	9 (0.3)	5 (0.2)
Cardiac disorders	6 (0.2)	2 (0.1)
Eye disorders	5 (0.2)	3 (0.1)
Ear and labyrinth disorders	4 (0.1)	2 (0.1)
Endocrine disorders	4 (0.1)	4 (0.2)
Musculoskeletal and connective tissue disorders	3 (0.1)	3 (0.1)
Renal and urinary disorders	3 (0.1)	1 (0.0)
Blood and lymphatic system disorders	2 (0.1)	2 (0.1)
Neoplasms benign, malignant and unspecified (incl. cysts and polyps)	2 (0.1)	2 (0.1)
Investigations	1 (0.0)	1 (0.0)
**Antipyretic medication use (prophylactic and treatment), *n* (%)**		
Yes	413 (13.5)	302 (13.7)
No	2650 (86.5)	1907 (86.3)

* In free text a non-binary gender identity was described.

**Table 2:** Characteristics of households of participants after baseline and questionnaire 1.

Characteristics of Households of Participants		
	HPV Dose 1 (*n* = 3063)	HPV Dose 2
		(*n* = 2209)
**Children per household, *n* (%)**		
One child	379 (12.4)	274 (12.4)
Two children	1827 (59.6)	1331 (60.3)
Three children	739 (24.1)	525 (23.8)
Four children	97 (3.2)	63 (2.9)
Five or more children	19 (0.6)	14 (0.6)
Information not available	2 (0.1)	2 (0.1)
**Education parent or guardian of participant, *n* (%)**		
Primary school	3 (0.1)	1 (0.0)
Pre-vocational secondary education	46 (1.5)	33 (1.5)
General secondary education	49 (1.6)	34 (1.5)
Vocational education	463 (15.1)	335 (15.2)
University of applied sciences	1337 (43.7)	967 (43.8)
University	1145 (37.4)	830 (37.6)
PhD/Postdoc	9 (0.3)	3 (0.1)
Information not available	11 (0.4)	6 (0.3)
**After-school care, *n* (%)**		
Yes	1617 (52.8)	1184 (53.6)
No	1446 (47.2)	1025 (46.4)

**Table 3:** AEFI reported after HPV vaccination (*n*, %).

	HPV Dose 1 (*n* = 3063)	HPV Dose 2 (*n* = 2209)
At least one AEFI	1812 (59.2)	914 (41.4)
At least one pre-defined (checklist) AEFI	1598 (52.2)	797 (36.1)
At least one other AEFI	516 (16.8)	212 (9.6)
At least one serious AEFI	0 (0.0)	0 (0.0)

**Table 4:** Solicited AEFI reported after HPV vaccination (*n*, %).

Solicited AEFI (MedDRA Preferred Term)		
	HPV Dose 1 (*n* = 3063)	HPV Dose 2 (*n* = 2209)
Injection site reaction	1424 (46.5)	704 (31.9)
Headache	252 (8.2)	86 (3.9)
Arthralgia	137 (4.5)	81 (3.7)
Pyrexia	108 (3.5)	43 (1.9)
Nausea	98 (3.2)	35 (1.6)
Rash	43 (1.4)	8 (0.4)
Vomiting	31 (1.0)	10 (0.5)

**Table 5:** Other non-solicited AEFI reported after HPV vaccination (*n*, %).

Top 10 other AEFI (MedDRA Preferred Term)		
	HPV Dose 1 (*n* = 3063)	HPV Dose 2 (*n* = 2209)
Fatigue	92 (3.0)	25 (1.1)
Myalgia	90 (2.9)	60 (2.7)
Pain in extremity	82 (2.7)	37 (1.7)
Limb discomfort	52 (1.7)	35 (1.6)
Abdominal pain	42 (1.4)	9 (0.4)
Nasopharyngitis	35 (1.1)	8 (0.4)
Body temperature increased	24 (0.8)	4 (0.2)
Malaise	24 (0.8)	10 (0.5)
Oropharyngeal pain	22 (0.7)	5 (0.2)
Dizziness	19 (0.6)	7 (0.3)

## Data Availability

The data from the CEM study cannot be made fully publicly available due to the General Data Protection Regulation (GDPR) and the general privacy regulation of Pharmacovigilance Centre Lareb. Request to access the data may be granted on reasonable request via the corresponding author.

## Supplementary Materials

The following supporting information can be downloaded at: https://www.mdpi.com/article/10.3390/vaccines13080812/s1. Supplementary Materials A—List of all reported AEFI in the study; Supplementary Materials B—Time to onset (TTO) and Time to Recovery (TTR) for the top 10 of non-solicited AEFI after HPV vaccination (1st and 2nd dose); Supplementary Materials C—Burden for the top 10 of non-solicited AEFI after HPV vaccination (1st and 2nd dose); Supplementary Materials D—Re-occurrence risk based on (corrected) risk ratios.

## Abbreviations

The following abbreviations are used in this manuscript:

AEFI	Adverse event following immunization
CEM	Cohort event monitoring
CIOMS	Council for International Organizations of Medical Sciences
HPV	Human papillomavirus
Lareb	The Dutch Pharmacovigilance Centre
MeDRA	Medical Dictionary for Regulatory Activities
NIP	National Immunization Program
PRO	Patient Reported Outcome
RIVM	National Institute for Public Health and the Environment
TTO	Time to onset
