# Supplementary material for: Frequency, Timing, Burden and Recurrence of Adverse Events Following Immunization After HPV Vaccine Based on a Cohort Event Monitoring Study in the Netherlands
Source: Vaccines (Basel). 2025 Jul 30;13(8):812. doi: 10.3390/vaccines13080812 (PMC12389782; doi:10.3390/vaccines13080812)
Supplement: Supplementary file 1 [file vaccines-13-00812-s001.zip › vaccines-3759720-supplementary.pdf]

Supplementary Materials A List of all reported AEFI in the study

Supplementary Materials A.1 All reported AEFI (MedDRA Preferred Term) for the 1st HPV vaccination

AEFI	*n*	%
Injection site reaction	14241,424	64.5
Headache	252	11.4
Arthralgia	137	6.2
Pyrexia	108	4.9
Nausea	98	4.4
Fatigue	92	4.2
Myalgia	90	4.1
Pain in extremity	82	3.7
Limb discomfort	52	2.4
Rash	43	1.9
Abdominal pain	42	1.9
Nasopharyngitis	35	1.6
Vomiting	31	1.4
Malaise	24	1.1
Body temperature increased	24	1.1
Oropharyngeal pain	22	1
Dizziness	19	0.9
Cough	18	0.8
Listless	18	0.8
Influenza like illness	13	0.6
Syncope	8	0.4
Injected limb mobility decreased	7	0.3
Rash erythematous	7	0.3
Diarrhoea	6	0.3
Extensive swelling of vaccinated limb	6	0.3
Pallor	6	0.3
Rash pruritic	6	0.3
Insomnia	5	0.2
Erythema infectiosum	5	0.2
Decreased appetite	4	0.2
Haematoma	4	0.2
Musculoskeletal stiffness	3	0.1
Impetigo	3	0.1
Crying	3	0.1
Eczema	3	0.1
Chest pain	3	0.1
Dyspnoea	3	0.1
Eye pruritus	3	0.1
Mood altered	3	0.1
Ear pain	2	0.1
Pruritus	2	0.1
Chills	2	0.1

Epistaxis	2	0.1
Feeling cold	2	0.1
Pain	2	0.1
Cystitis	2	0.1
Sneezing	2	0.1
Abdominal discomfort	2	0.1
Asthenia	1	0
Pneumonia	1	0
Muscular weakness	1	0
Nasal congestion	1	0
Visual impairment	1	0
Hyperhydrosis	1	0
Muscle spasms	1	0
Neck pain	1	0
Papule	1	0
Rhinorrhoea	1	0
Somnolence	1	0
Constipation	1	0
Discomfort	1	0
Nightmare	1	0
Oral herpes	1	0
Rash papular	1	0
Urticaria	1	0
Anxiety	1	0
Blister	1	0
Feeling hot	1	0
Glossodynia	1	0
Myringitis	1	0
Oedema	1	0
Pollakiuria	1	0
Pustule	1	0
Respiratory tract infection	1	0
Scarlet fever	1	0
Sensory overload	1	0
Stomatitis	1	0
Vulvovaginal pain	1	0

Supplementary Materials A.2 All reported AEFI (MedDRA Preferred Term) for the 2nd HPV vaccination

AEFI	*n*	%
Injection site reaction	704	31.9
Headache	86	3.9
Arthralgia	81	3.7
Myalgia	60	2.7
Pyrexia	43	1.9
Pain in extremity	37	1.7
Nausea	35	1.6

Limb discomfort	35	1.6
Fatigue	25	1.1
Vomiting	10	0.5
Malaise	10	0.5
Abdominal pain	9	0.4
Nasopharyngitis	8	0.4
Rash	8	0.4
Dizziness	7	0.3
Oropharyngeal pain	5	0.2
Pain	5	0.2
Injected limb mobility decreased	4	0.2
Body temperature increased	4	0.2
Extensive swelling of vaccinated limb	4	0.2
Listless	3	0.1
Syncope	2	0.1
Pallor	2	0.1
Lymphadenopathy	2	0.1
Herpes zoster	2	0.1
Cough	1	0
Diarrhoea	1	0
Influenza like illness	1	0
Insomnia	1	0
Impetigo	1	0
Pruritus	1	0
Irritability	1	0
Epistaxis	1	0
Feeling cold	1	0
Papule	1	0
Discomfort	1	0
Dyspnoea	1	0
Emotional disorder	1	0
Hypersensitivity	1	0
Oral herpes	1	0
Pharyngitis	1	0
Taste disorder	1	0
Tic	1	0
Glossodynia	1	0
Epididymitis	1	0
Fungal skin infection	1	0
Otitis media	1	0
Testicular pain	1	0

Supplementary Materials B

Time to onset (TTO) and Time to Recovery (TTR) for the top 10 of non-solicited AEFI after HPV vaccination (1st and 2nd dose)

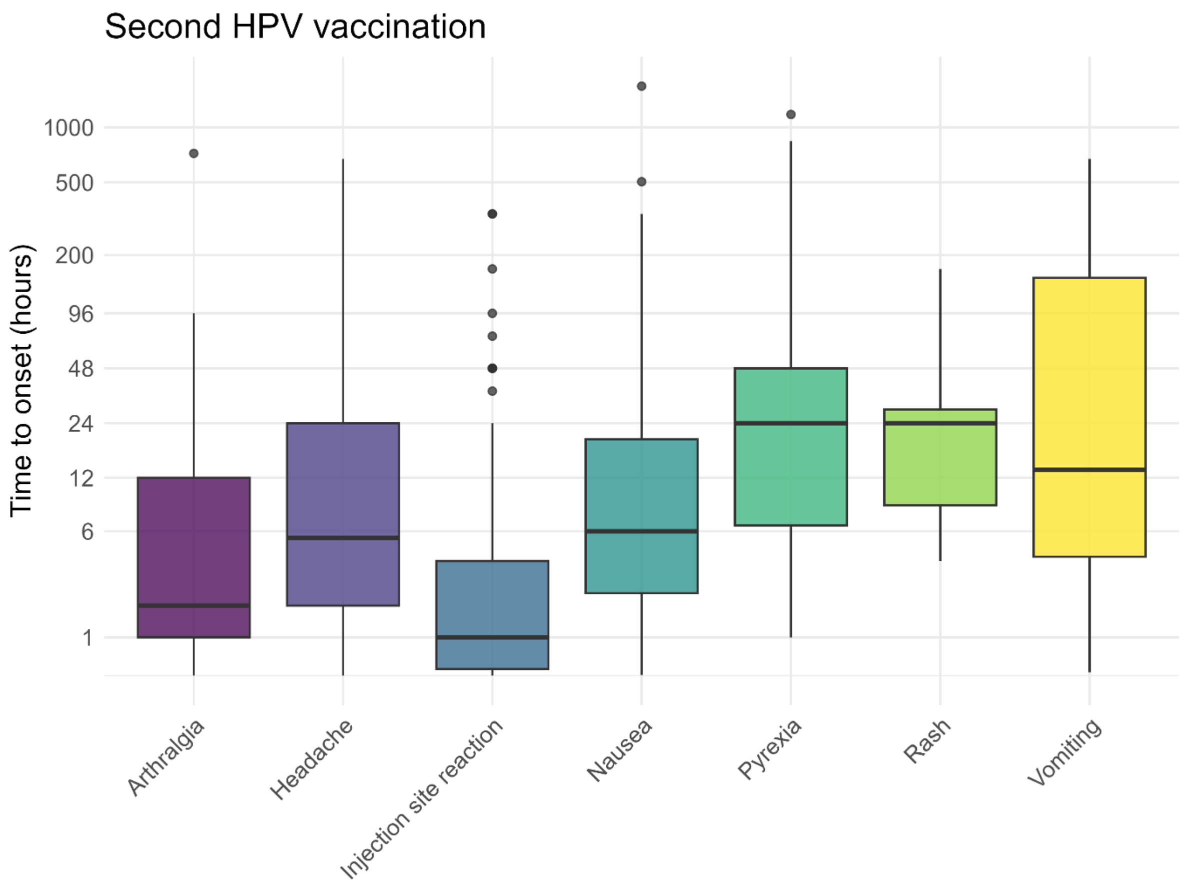

Figure S1. Time to onset for non-solicited AEFI after the 1st HPV vaccination , dots represent outliers.

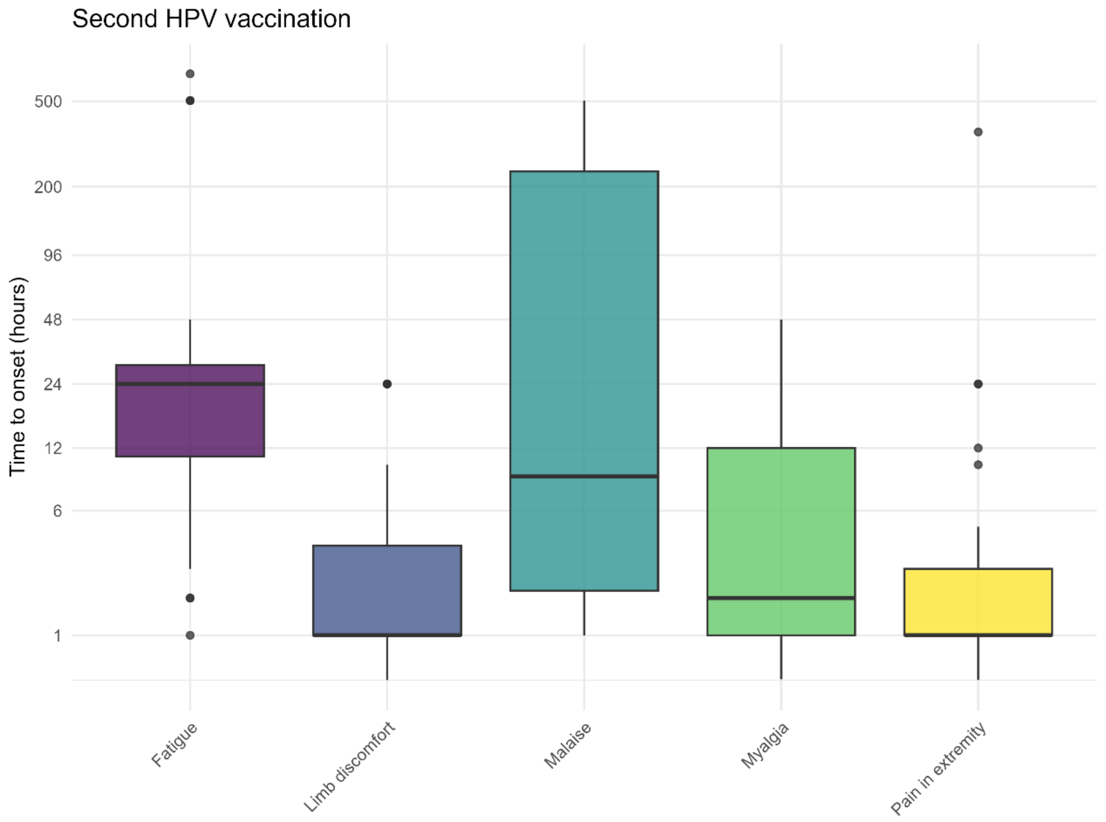

Figure S2. Time to onset for non-solicited AEFI after the 2nd HPV vaccination, dots represent outliers.

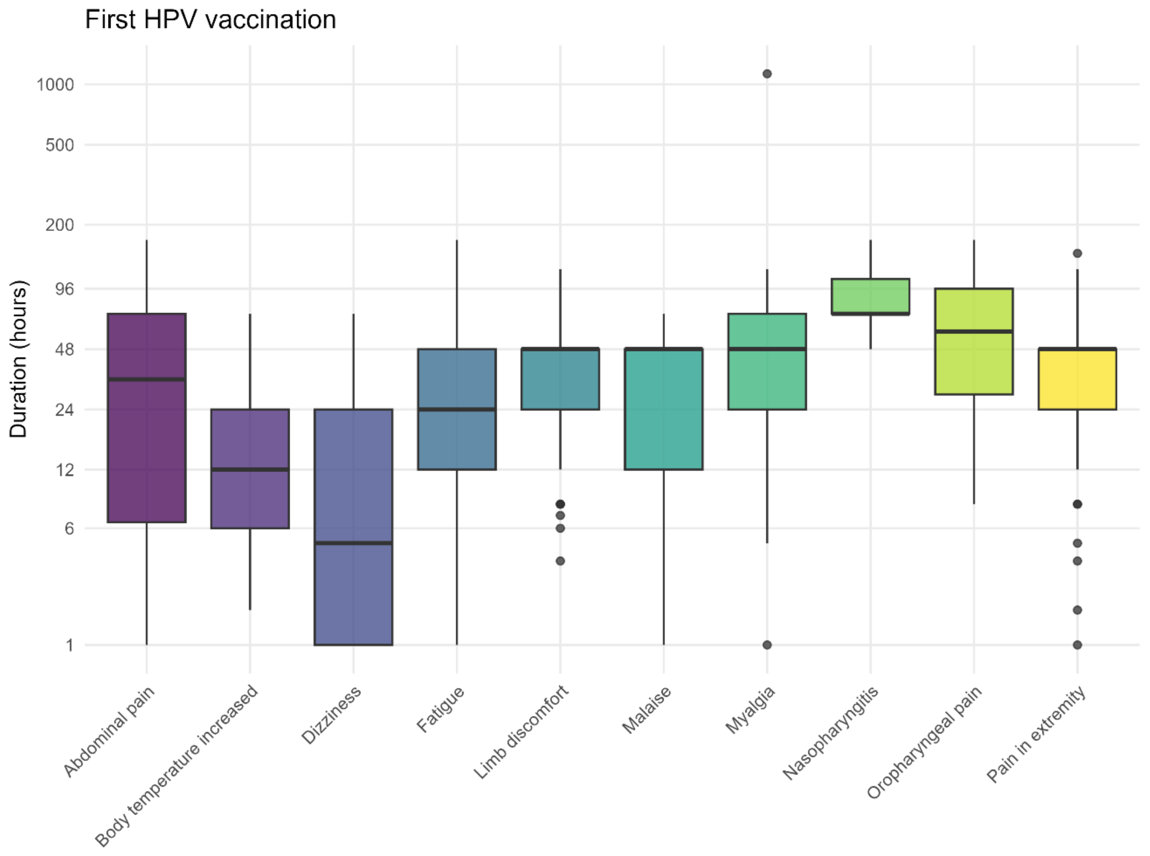

Figure S3. Duration for non-solicited AEFI after the 1st HPV vaccination, dots represent outliers.

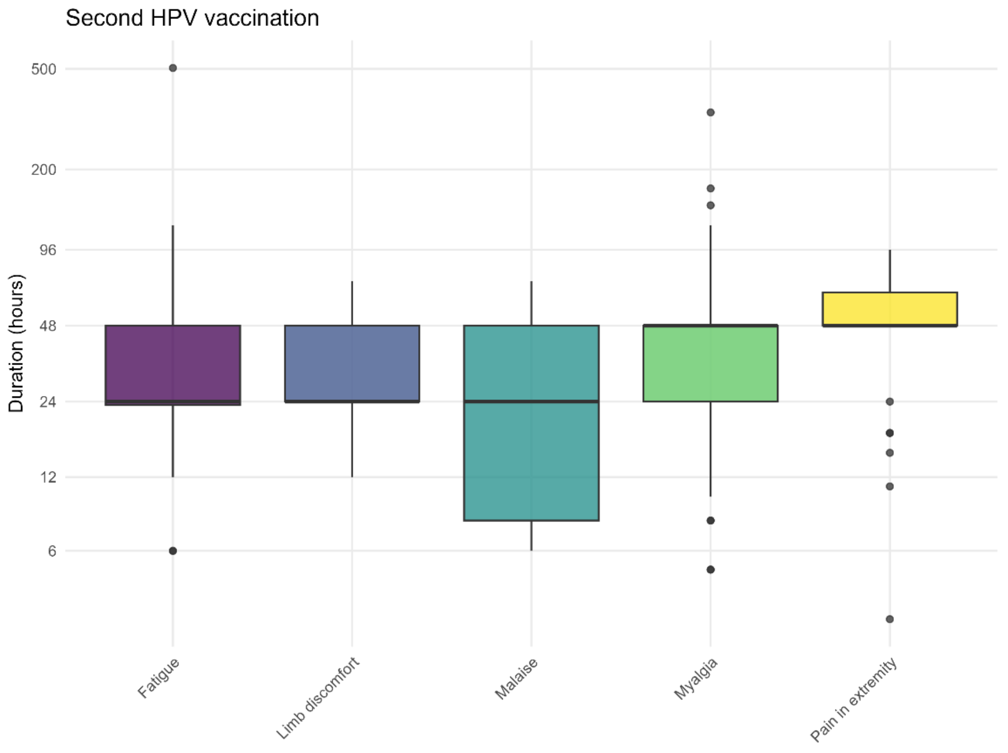

Figure S4. Duration for non-solicited AEFI after the 2nd HPV vaccination, dots represent outliers.

Supplementary Materials C Burden for the top 10 of non-solicited AEFI after HPV vaccination (1st and 2nd dose)

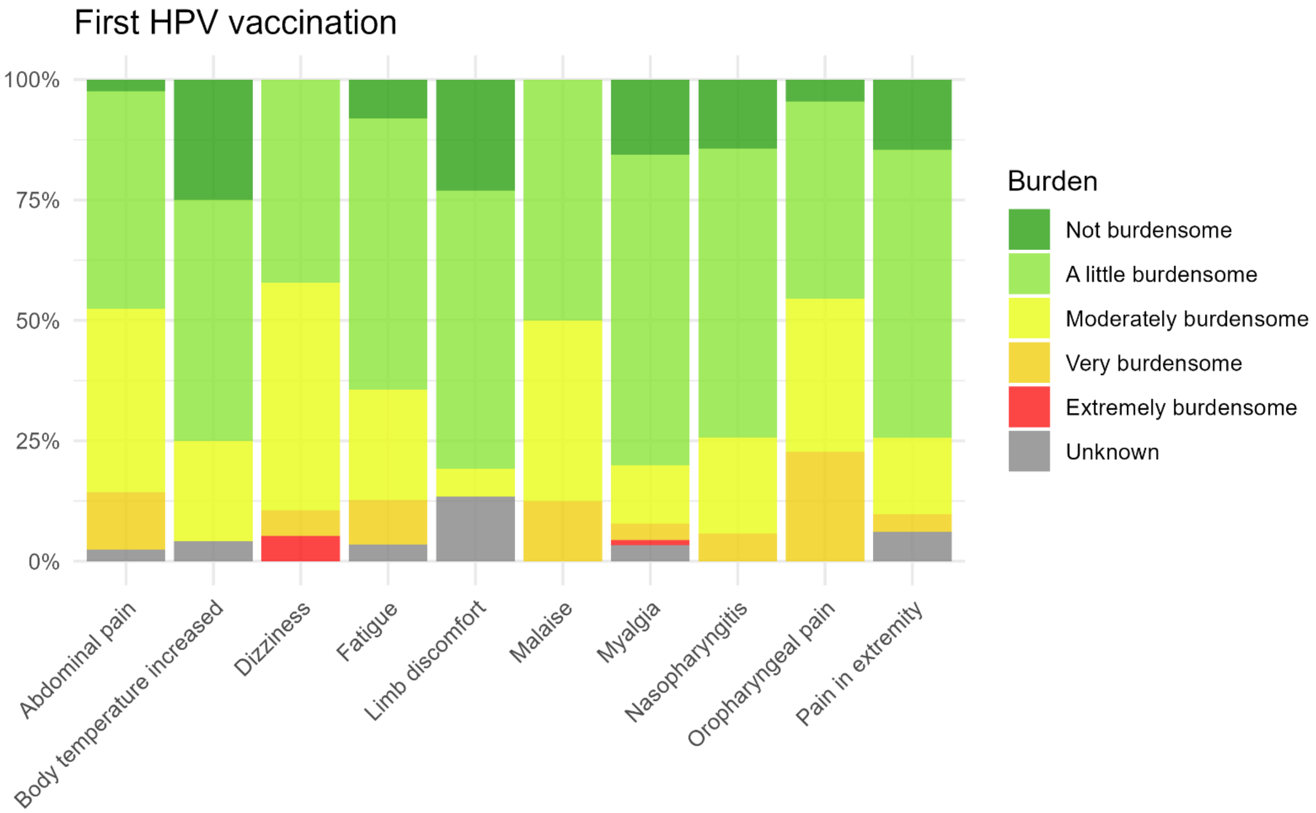

Figure S5. Burden for non-solicited AEFI after the 1st HPV vaccination

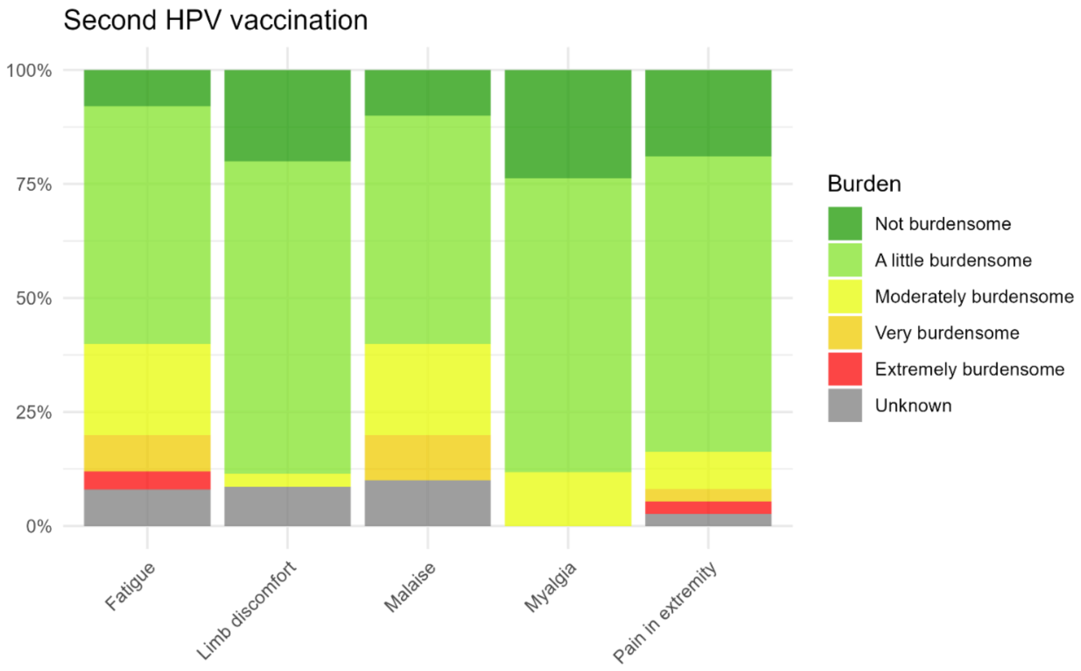

Figure S6. Burden for non-solicited AEFI after the 2nd HPV vaccination

Supplementary Materials D Recurrence risk based on (corrected) risk ratios

Risk ratio was corrected for: the child's sex, attendance at after-school care, and whether the child lived in a household with multiple children.

AEFI after dose 1 and 2							Crude Risk Ratio	Corrected Risk Ratio		
AEFI			AEFI after 2nd vaccination			AEFI after 2nd vaccination = yes				
			Yes	No	a+c	Risk (%)	Risk Ratio (95%CI)		RR	95%CI
Arthralgia	AEFI after 1st vaccination	Yes	16	71	80	18,39	6,09 (3,54 - 9,80)	Arthralgia	6,18	3,74 - 10,24
		No	64	2058		3,02		Sex (1 = boy)	1,35	0,88 - 2,07
								Multiple children	1	0,52 - 1,90
								After school care	0,82	0,54 - 1,25
Fatigue	AEFI after 1st vaccination	Yes	1	58	25	1,69	1,52 (0,09 - 6,98)	Fatigue	1,47	0,20 - 10,75
		No	24	2126		1,12		Sex (1 = boy)	0,98	0,45 - 2,15
								Multiple children	1,04	0,32 - 3,48
								After school care	0,68	0,31 - 1,50
Headache	AEFI after 1st vaccination	Yes	24	123	84	16,33	5,61 (3,53 - 8,61)	Headache	5,34	3,42 - 8,33
		No	60	2002		2,91		Sex (1 = boy)	0,84	0,55 - 1,28
								Multiple children	0,66	0,40 - 1,11
								After school care	0,67	0,44 - 1,01
Injection site reactions	AEFI after 1st vaccination	Yes	499	510	704	49,45	2,98 (2,52 - 3,34)	Injection site reactions	2,88	2,51 - 3,31
		No	205	995		17,08		Sex (1 = boy)	0,84	0,75 - 0,95
								Multiple children	0,97	0,83 - 1,14
								After school care	1,03	0,92 - 1,16
Limb discomfort	AEFI after 1st vaccination	Yes	2	37	30	5,13	3,84 (0,63 - 12,11)	Limb discomfort	3,36	0,82 - 13,7
		No	28	2141		1,29		Sex (1 = boy)	0,58	0,28 - 1,21
								Multiple children	2,11	0,51 - 8,74
								After school care	1,35	0,66 - 2,77
Myalgia	AEFI after 1st vaccination	Yes	13	43	59	23,21	10,87 (5,94 - 18,30)	Myalgia	10,79	6,17 - 18,89
		No	46	2107		2,14		Sex (1 = boy)	1,03	0,63 - 1,69
								Multiple children	1,18	0,53 - 2,61
								After school care	1,15	0,70 - 1,89
Nausea	AEFI after 1st vaccination	Yes	6	54	35	10,00	7,41 (2,86 - 15,93)	Nausea	6,62	2,86 - 15,30
		No	29	2120		1,35		Sex (1 = boy)	0,52	0,26 - 1,06

								Multiple children	0,34	0,17 - 0,69
								After school care	0,7	0,36 - 1,34
Pain in extremities	AEFI after 1st vaccination	Yes	2	62	35	3,13	2,03 (0,33 - 6,47)	Pain in extremities	2,04	0,50 - 8,36
		No	33	2112		1,54		Sex (1 = boy)	0,89	0,46 - 1,73
								Multiple children	0,85	0,33 - 2,18
								After school care	1,02	0,53 - 1,98
Pyrexia	AEFI after 1st vaccination	Yes	4	64	43	5,88	3,23 (0,99 - 7,74)	Pyrexia	2,94	1,08 - 8,03
		No	39	2102		1,82		Sex (1 = boy)	0,98	0,54 - 1,78
								Multiple children	0,42	0,21 - 0,83
								After school care	0,63	0,35 - 1,16

Factors that significantly contribute to the recurrence risk for any of the reported complaints are shown in green
